# Synthesis of silver nanoparticles using reducing agents obtained from natural sources (*Rumex hymenosepalus* extracts)

**DOI:** 10.1186/1556-276X-8-318

**Published:** 2013-07-10

**Authors:** Ericka Rodríguez-León, Ramón Iñiguez-Palomares, Rosa Elena Navarro, Ronaldo Herrera-Urbina, Judith Tánori, Claudia Iñiguez-Palomares, Amir Maldonado

**Affiliations:** 1 Departamento de Investigación en Física, Universidad de Sonora, Hermosillo, Sonora 83000, México; 2 Departamento de Ingeniería Química y Metalurgia, Universidad de Sonora, Hermosillo, Sonora 83000, México; 3 Departamento de Investigación en Polímeros y Materiales, Universidad de Sonora, Hermosillo, Sonora 83000, México; 4 Departamento de Física, Universidad de Sonora, Apartado Postal 1626, Hermosillo, Sonora 83000, México; 5 Centro de Investigación en Alimentación y Desarrollo A.C, Hermosillo, Sonora 83304, México

**Keywords:** Silver nanoparticles, *Rumex hymenosepalus*, Antioxidants, Electron microscopy, Green synthesis

## Abstract

We have synthesized silver nanoparticles from silver nitrate solutions using extracts of *Rumex hymenosepalus*, a plant widely found in a large region in North America, as reducing agent. This plant is known to be rich in antioxidant molecules which we use as reducing agents. Silver nanoparticles grow in a single-step method, at room temperature, and with no addition of external energy. The nanoparticles have been characterized by ultraviolet-visible spectroscopy and transmission electron microscopy, as a function of the ratio of silver ions to reducing agent molecules. The nanoparticle diameters are in the range of 2 to 40 nm. High-resolution transmission electron microscopy and fast Fourier transform analysis show that two kinds of crystal structures are obtained: face-centered cubic and hexagonal.

## Background

The synthesis of nanomaterials is of current interest due to their wide variety of applications in fields such as electronics [1-4], photonics [5-7], catalysis [8-10], medicine [11-15], etc. Most of the applications are due to the fact that matter at the nanometer scale has different properties as compared with the bulk state. For this reason, many research groups around the world are trying new methods of synthesis of different materials at the nanoscale. One goal is to control the size and shape of atomic clusters or nanoparticles and their ordering in 1D, 2D, or 3D arrays. In particular, silver nanoparticles have been used with promising results as bactericides [16-21], antimicotics [22], and anticancer agents [21,23,24].

Several methods have been devised in order to prepare metallic nanoparticles. For instance, one of the current methods crystalizes nanoparticles in microemulsions, using a variety of chemicals as precursors and large amounts of surfactants as stabilizing agents. The different preparation methods have been successful in the synthesis of nanoparticles of several materials: metallic [25-27], dielectric [28,29], semiconductor [30,31], and magnetic [32,33]. However, the intensive use of solvents and synthetic reactants is harmful for the environment. For this reason, it is very desirable to devise alternative, ‘green’ methods of nanomaterial preparation that use environmentally friendly reactants. The silver nanoparticles obtained by the green synthesis method are candidates to be used in biological systems.

In the case of silver particles, the nanocrystals are usually grown from Ag^+^ solutions. The silver ions come from a salt like silver nitrate (AgNO_3_). The ions are first reduced to atoms by means of a reducing agent. The obtained atoms then nucleate in small clusters that grow into particles. Depending on the availability of atoms, which in turn depends on the silver salt to reducing agent concentration ratio, the size and shape of the nanoparticles can be controlled. In this method, two elements are needed for the nanoparticle grow: a silver salt and a reducing agent [34,35].

On the other hand, in recent times, there is a growing interest in the synthesis of metal nanoparticles by ‘green’ methods. For this purpose, biomass or extracts of different plants have been tried with success as reducing agents. For instance, in the literature, there are reports of the synthesis of silver or gold nanoparticles using extracts of different plants [17-20,23,24,36-49]. The present work is part of this new line of research.

In our study, the reducing agent comes from extracts of *Rumex hymenosepalus*, which is a plant rich in polyphenols. In the literature, there is no report on the synthesis of nanoparticles using extracts from this plant. It is a vegetal species abundantly present in North Mexico and in the south of the USA. In Mexico, it is collected, dried, cut, and packed for selling to the public. This plant, also known as *canaigre dock* or *wild rhubarb*, can be of interest for green synthesis because it contains a large amount of natural antioxidants. Among the antioxidant molecules this plant contains, polyphenolic compounds, like flavan-3-ols (tannins) and stilbenes, are found in large quantities. These molecules are potentially strong reducing agents due to their numerous OH groups that promote their antioxidant activity [50,51].

In this paper, we present results on the synthesis of silver nanoparticles using extracts of the plant *R. hymenosepalus* (Rh extracts) as reducing agent in aqueous silver nitrate solutions. We have extracted the antioxidant fractions from dried roots of the plant. We have characterized the resulting nanoparticles by transmission electron microscopy (TEM) and ultraviolet-visible (UV-Vis) spectroscopy. To the best of our knowledge, this is the first report in the literature on nanoparticle synthesis using extracts of this plant.

## Methods

We have purchased dried, slice-cut roots of *R. hymenosepalus* in a local convenient store (Comercial Zazueta, Hermosillo, Mexico); we present a picture of the dried roots in the Additional file 1: Figure S1. Ethanol (99%) and silver nitrate (AgNO_3_ 99%) are from Sigma-Aldrich (St. Louis, MO, USA). For the UV-Vis calibration curves, we have used epicatechin (98%) and epicatechin gallate (95%); both molecules were purchased in Sigma-Aldrich. We have used ultra-purified water (Milli Q system, Millipore, Billerica, MA, USA).

In order to prepare the plant extract, we have put 15 g of a dried *R. hymenosepalus* sample in a flask, and then, we have added 100 ml of an ethanol/water solution (70:30 *v*/*v*). The flask was stored at room temperature (*T* = 25°C), and the extraction was allowed to proceed during several days; the visual appearance of the liquid was monitored on a daily basis. Its color changed from a light red the first day to a darker brown. After 15 days, the extraction was considered complete since no change in the color was noticeable. The sample was then filtered, and the resulting liquid is the Rh (*R. hymenosepalus*) extract that has been used as reducing agent in the nanoparticle synthesis. The Rh extract has been characterized by UV-Vis spectroscopy (Perkin Elmer Lambda 20 spectrophotometer, PerkinElmer, Waltham, MA, USA) and proton nuclear magnetic resonance (^1^H NMR) experiments with a Bruker Avance 400 apparatus (Bruker AXS Inc., Madison, WI, USA) operating at 400 MHz, at 25°C. For the NMR experiments, a portion of the Rh extract was concentrated on a rotary evaporator at 37°C and dried under vacuum. The resulting dark brown solid was washed three times with 100 ml of tetrahydrofuran (Aldrich 99.9% purity) and purified using a glass filter. The filtrate was evaporated and dried under vacuum. With the solid, obtained NMR tubes were prepared in deuterated dimethyl sulfoxide (DMSO-*d*_6_). The internal reference was tetramethylsilane.

For the nanoparticle synthesis, we have prepared one solution of AgNO_3_ in water; the concentration was 0.1 M. Different volumes of this solution have been mixed with a fixed volume of the Rh extract (*V*_Rh_ = 200 μl); the total volume of each sample was adjusted to 4 ml by adding the necessary amount of ethanol in order to prepare samples with different AgNO_3_ concentrations: 2.5, 5, 7.5, 10, and 15 mM. The extract concentration was 5% *v*/*v* in all the samples.

For each AgNO_3_ concentration, the reduction reaction has proceeded along 96 h. The experiment was performed under regular, indoor illumination. The samples were analyzed every 24 h by visual inspection and UV-Vis spectroscopy. The nanoparticles have been observed with TEM using a Jeol 2010 F apparatus (JEOL Ltd., Akishima-shi, Japan) operating at 200 kV. We have deposited 10 μl of the nanoparticle suspension on a formvar-carbon coated copper TEM grid (300 Mesh). The sample was vacuum-dried for 24 h before observation. From the TEM micrographs, the size distribution was obtained, as well as the average diameter. The chemical composition of the nanoparticles has been obtained with energy dispersive X-ray spectroscopy (EDS) using a Bruker Quantax 200 detector (Bruker AXS Inc., Madison, WI, USA). The crystal structure of the nanoparticles has been obtained from high-resolution TEM (HR-TEM) experiments and from the corresponding fast Fourier transform (FFT) plots.

## Results and discussion

The extraction procedure from dried *R. hymenosepalus* roots yielded a dark liquid which we examined spectroscopically. The UV-Vis and NMR experiments confirm the presence of polyphenols in the Rh extract. The absorbance of the sample presented a peak in the range of 277 to 280 nm as characteristic of polyphenol molecules. On the other hand, the ^1^H NMR proton spectra display a wealth of peaks characteristic of plant extracts (Additional file 1: Figure S2). We have identified some of these signals as corresponding to polyphenol molecules [52] (Additional file 1: Figures S3 and S4). In particular, some peaks correspond to catechines and stilbene molecules. For instance, at least five chemical shifts of our spectra match those of epicatechin, as reported in the SDBS spectral database of organic compounds (no. 22007HSP-44-526). The coincidences are shown in Additional file 1: Table S1. The chemical shifts also match those reported for epicatechin gallate and epigallocatechin gallate (Additional file 1: Table S1). In the Additional file 1: Figure S5, we display the chemical structure of these molecules. On the other hand, ten of the peaks match those reported for a stilbene compound extracted from roots of the *Terminalia sericeae* tree [53] (Additional file 1: Table S1). These signals correspond to a stilbene molecule known as stilbene glycoside (Additional file 1: Figure S6). The NMR results obtained so far allow us to assess a significant presence of polyphenolic compounds in the plant extract of *R. hymenosepalus*. These compounds are potential reductor agents in the synthesis mechanism of silver nanoparticles. From UV-Vis calibration curves (using pure compounds), we estimate the concentration of two of the reducing molecules: epicatechin (241 μM) and epicatechin gallate (91.1 μM). Additional NMR experiments are under way in order to further characterize this plant extract. The results will be published elsewhere.

Since the *R. hymenosepalus* extract contains polyphenols, we can anticipate that it will serve as reducing agent for the nanoparticle synthesis. In fact, the same molecular mechanisms that give antioxidant properties to these molecules must promote the reduction of Ag^+^ ions to Ag atoms. The main mechanism is hydrogen abstraction [54] due to the OH groups in the polyphenol molecules.

**Figure 1:**
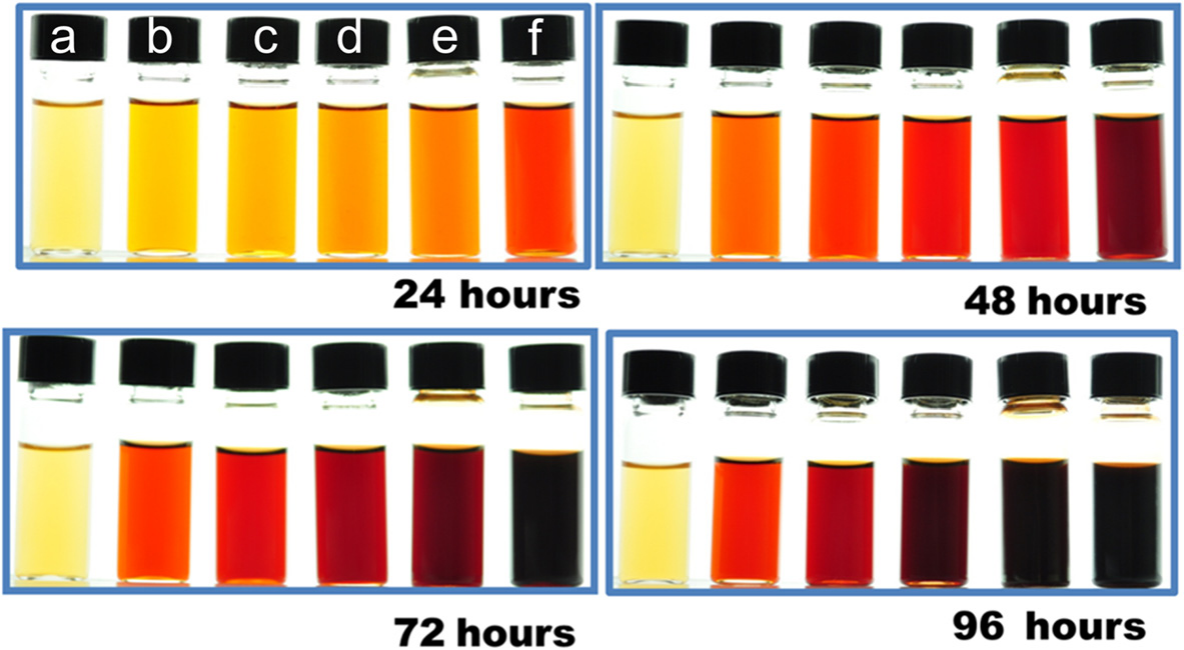
**Visual appearance of vials containing the *Rumex hymenosepalus *extract and AgNO**_**3 **_**solution after different reaction times.** The vials correspond to different AgNO_3_ concentrations: **(a)** pure extract, **(b)** 2.5 mM, **(c)** 5 mM, **(d)** 7.5 mM, **(e)** 10 mM, and **(f)** 15 mM. The change in color is an indication of the growth of silver nanoparticles.

We have thus prepared silver nanoparticles using the *R. hymenosepalus* extracts as reducing agent. For all the AgNO_3_ concentrations, the samples changed their visual appearance shortly after addition of the plant extract, indicating that a reduction reaction took place. Initially, the reacting mixture was a slightly yellowish liquid; as the reaction proceeded, the solutions became orange, red, and brown. This is a strong indication of the formation of silver nanoparticles: the change in color is due to the strong absorption of visible light due to excitation of the nanoparticle surface plasmons [55-58]. In Figure 1, we show vials with reacting samples for different AgNO_3_ concentrations (0, 2.5, 5, 7.5, 10, and 15 mM), and different times after the reaction started (24, 48, 72, and 96 h); the clear time evolution is a signal of the growth of silver nanoparticles. The time scale of the visual evolution depends on the AgNO_3_ concentration. For instance, for *C*_AgNO3_ = 5 mM, nanoparticle formation is visually appreciable after 4 min of the beginning of the reaction.

**Figure 2:**
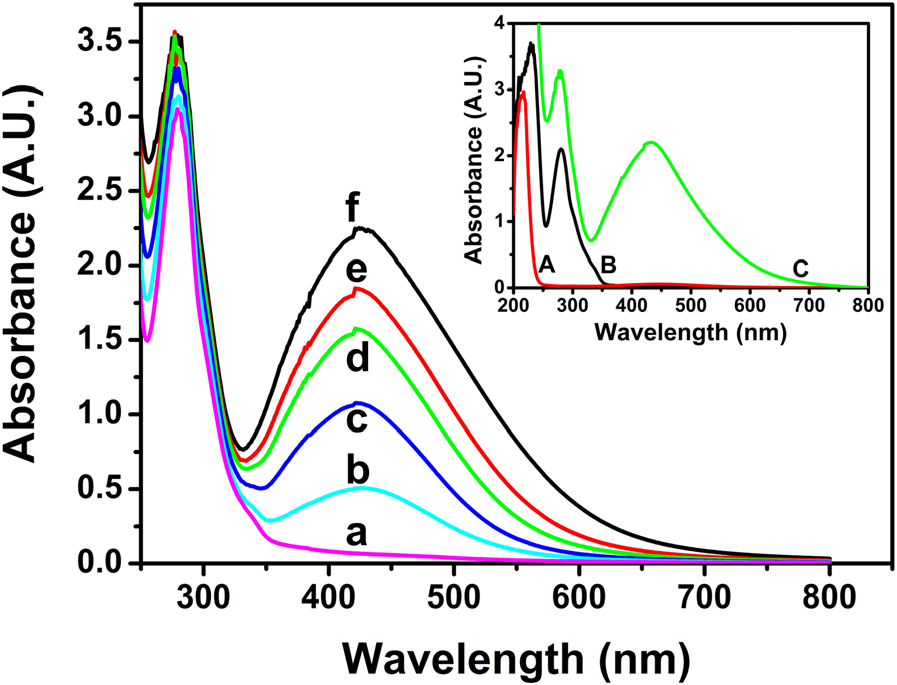
**UV–vis absorbance for samples with different values of AgNO**_**3 **_**concentrations. (a)** Pure extract, **(b)** 2.5 mM, **(c)** 5 mM, **(d)** 7.5 mM, **(e)** 10 mM, and **(f)** 15 mM. The peak around 425 nm corresponds to the absorbance due to surface plasmons in the silver nanoparticles. Note that peak intensity increases with the AgNO_3_ concentration and that the absorption due to the reducing agent (polyphenols from the extract) is observed around 278 nm. For comparison, in the inset, we display the absorption of the pure AgNO_3_ solution **(A)**, the plant extract **(B)**, and a sample where nanoparticles are growing **(C)**. The reaction time was 96 h.

The change in color, and thus the formation of silver nanoparticles, was confirmed by the UV-Vis experiments. In Figure 2, we show the spectra for a reaction time of 96 h. The curves display a pronounced peak around 425 nm, as expected from the plasmon resonance of silver nanoparticles. The UV-Vis peak is more pronounced for higher AgNO_3_ concentrations, indicating that more nanoparticles per unit volume are formed when this concentration increases. Note that in all the spectra displayed in Figure 2, the polyphenol peak (observed in the Rh extract) is also clearly visible around 278 nm. In the inset of Figure 2, we also display the UV-Vis spectra of the AgNO_3_ solution; it has a peak around 217 nm, as expected for Ag^+^ ions.

Note that we have performed control experiments in order to discard the action of ethanol and microorganisms as reducing agents. In the case of ethanol, the UV-Vis experiments show no significant Ag^+^ ions reduction when AgNO_3_ was dissolved, without Rh extract, in pure ethanol and in an ethanol/water mixture (see Additional file 1: Table S2 and Figure S7). On the other hand, we have verified the absence of microorganisms in the samples. We have performed aerobic plate count experiments for mold, yeast, and aerobic mesophilic bacteria [59,60], for the reacting sample where the silver nitrate concentration was 15 mM. In the case of the aerobic mesophilic bacteria test, we used plate count agar as culture medium; the sample was incubated at 35°C for 48 h. The results show that no mesophilic bacteria grow in the plate (see Additional file 1: Figure S8). In fact, the colony forming unit (CFU) is <1 CFU/ml. For the mold and yeast count test, we used potato dextrose agar; the sample was incubated at 25°C for 5 days. No mold or yeast was detected in the plate (the resulting CFU is <1 CFU/ml) (see Additional file 1: Figure S8). Thus, we can be sure that the synthesis of nanoparticles was performed under fully abiotic conditions.

**Figure 3:**
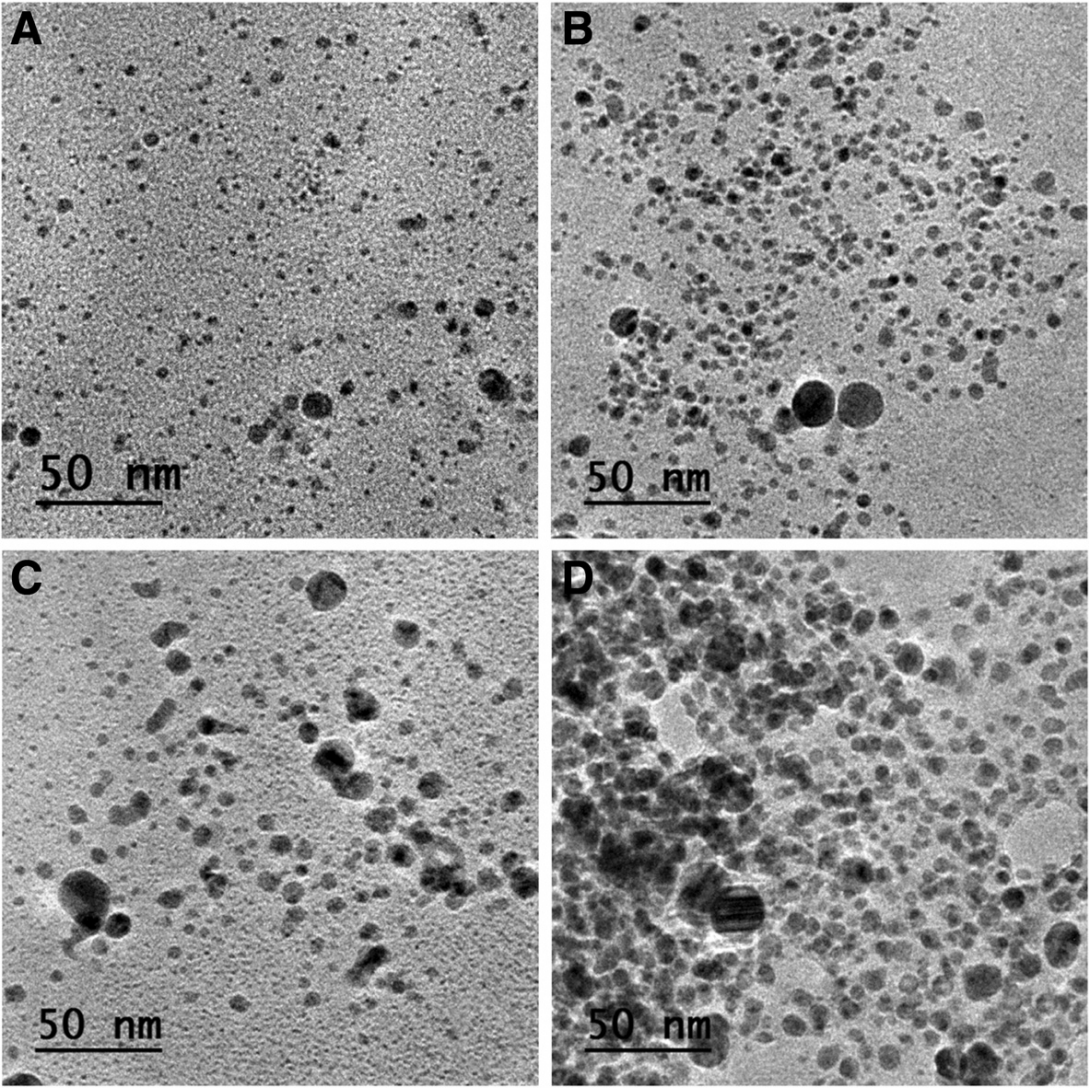
**TEM micrographs of the silver nanoparticles obtained for different AgNO**_**3 **_**concentrations. (A)** 2.5 mM, **(B)** 5 mM, **(C)** 7.5 mM, and **(D)** 15 mM, after a reaction time of 24 h.

**Figure 4:**
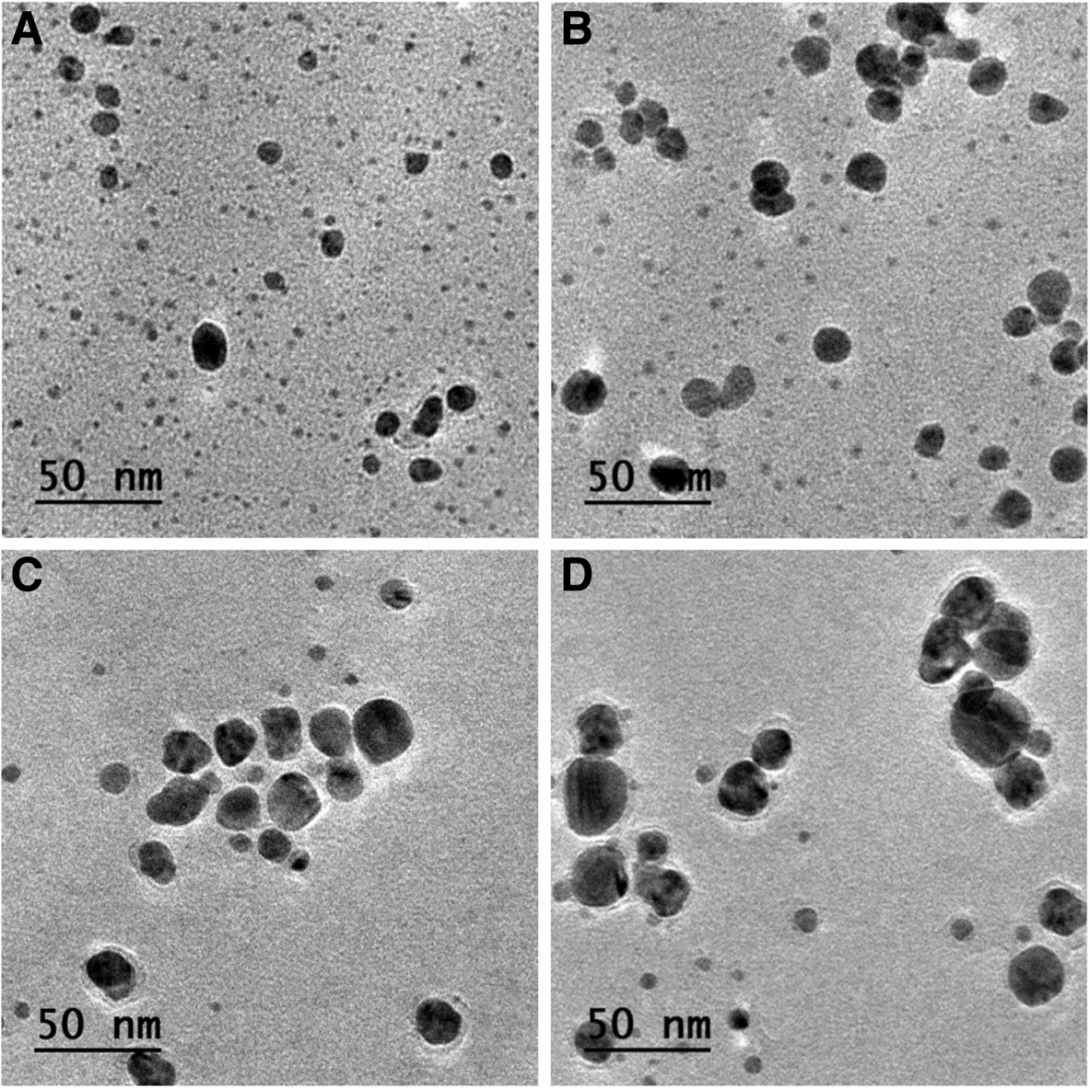
**TEM micrographs of the silver nanoparticles obtained for different AgNO**_**3 **_**concentrations. (A)** 2.5 mM, **(B)** 5 mM, **(C)** 7.5 mM, and **(D)** 15 mM, after a reaction time of 96 h.

We have characterized the silver nanoparticles with transmission electron microscopy. The size and abundance of the resulting particles depend on the AgNO_3_ concentration. Their diameter is in the range of 2 to 40 nm. In Figures 3 and 4, we present micrographs of the obtained silver nanoparticles after 24 and 96 h of the beginning of the reaction, for the different AgNO_3_ concentrations. For a reacting time of 24 h (Figure 3), we can appreciate that for *C*_AgNO3_ = 2.5 mM (micrograph A), the population is composed mainly of scattered, small nanoparticles. As the *C*_AgNO3_ increases, bigger nanoparticles are observed, while the proportion of small nanoparticles decreases. This trend is somehow maintained for a reacting time of 96 h (Figure 4). From the micrographs, we can observe that a population of big nanoparticles, in coexistence with a small proportion of small particles, is clearly appreciated. Furthermore, the size of the bigger particles increases as *C*_AgNO3_ is increased, while at the same time, the proportion of small nanoparticle decreases. Note that we do not observe particle coalescence, probably due to a stabilizing effect produced by the antioxidant molecules.

**Figure 5:**
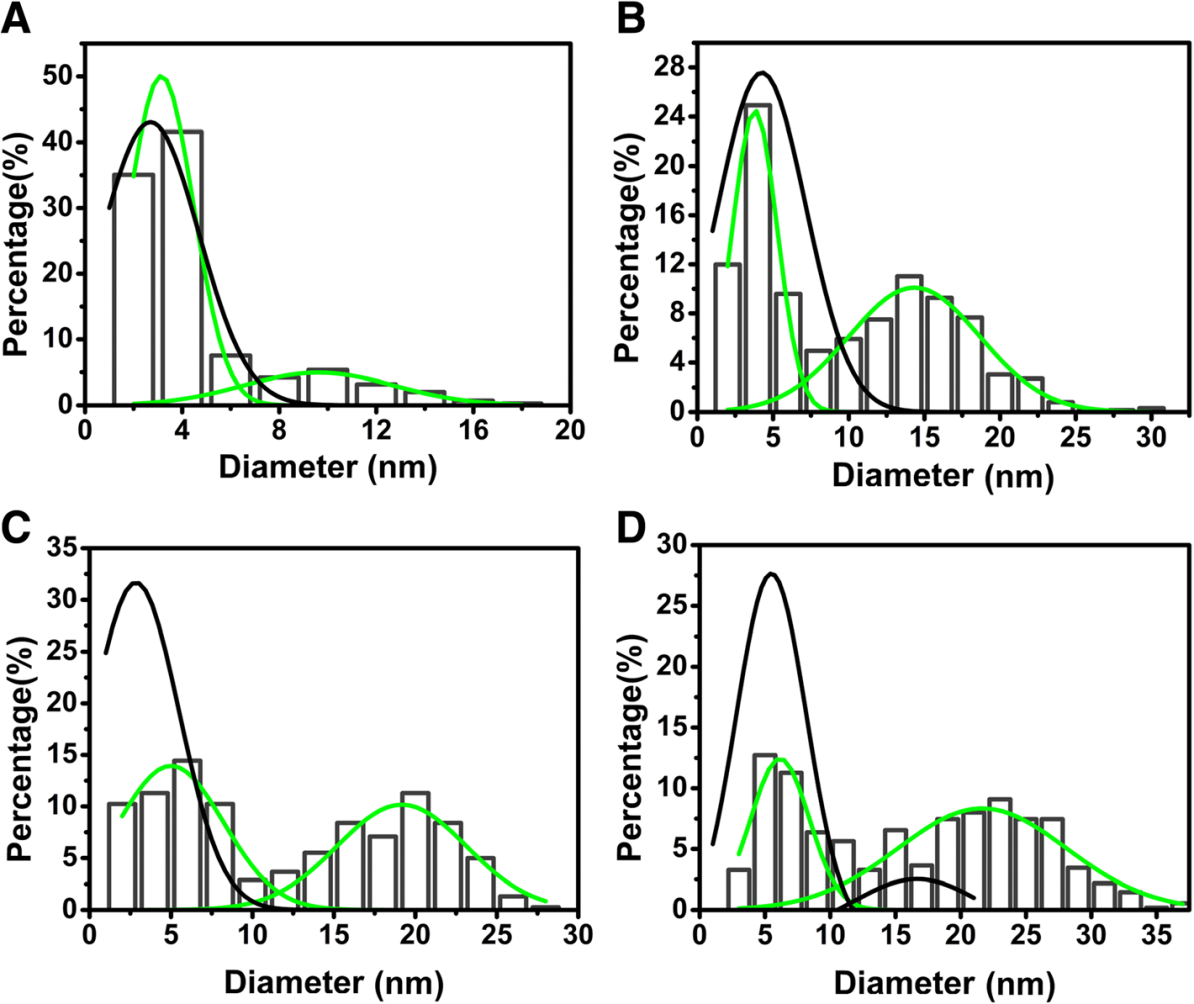
**Size distribution of the obtained silver nanoparticles for different values of the AgNO**_**3 **_**concentration. (A)** 2.5 mM, **(B)** 5 mM, **(C)** 7.5 mM, and (**D**) 15 mM, and two reaction times (24 and 96 h). For clarity, we display the full histogram and a fit (green curve) for 96 h, but only the fit (black curve) for 24 h. Note the two populations for a reaction time of 96 h. The statistical analysis has been performed with more than 500 nanoparticles in each case.

We have quantified these tendencies by statistically analyzing a population of more than 500 nanoparticles for each reaction time. The results are shown in Figure 5, where for matters of clarity, we present the full histograms for 96 h of reaction time, and only a representative curve for 24 h. For the shorter reaction time (24 h, black curves in Figure 5), most of the particles are small, with an average diameter around 3 to 5 nm. For 96 h after the beginning of the reaction, two populations are clearly distinguishable in the histograms. The first one is a subpopulation of small nanoparticles of average diameter around 4 to 5 nm. However, there exists also a considerable fraction of nanoparticles with larger average diameters, of the order of 10 to 20 nm. The average diameter of these larger particles grows with an increase in the AgNO_3_ concentration.

The chemical composition of the obtained particles was assessed by EDS spectroscopy. From the EDS spectra (see Additional file 1: Figure S9), we have confirmed that the nanoparticles are mainly composed of silver (subtracting the Cu, Si, and C contributions from the TEM grid and the detector window). Some amount of oxygen is also displayed in the EDS results (see Additional file 1: Table S3), probably meaning that some trace amount of the extract is still present in the TEM grid.

**Figure 6:**
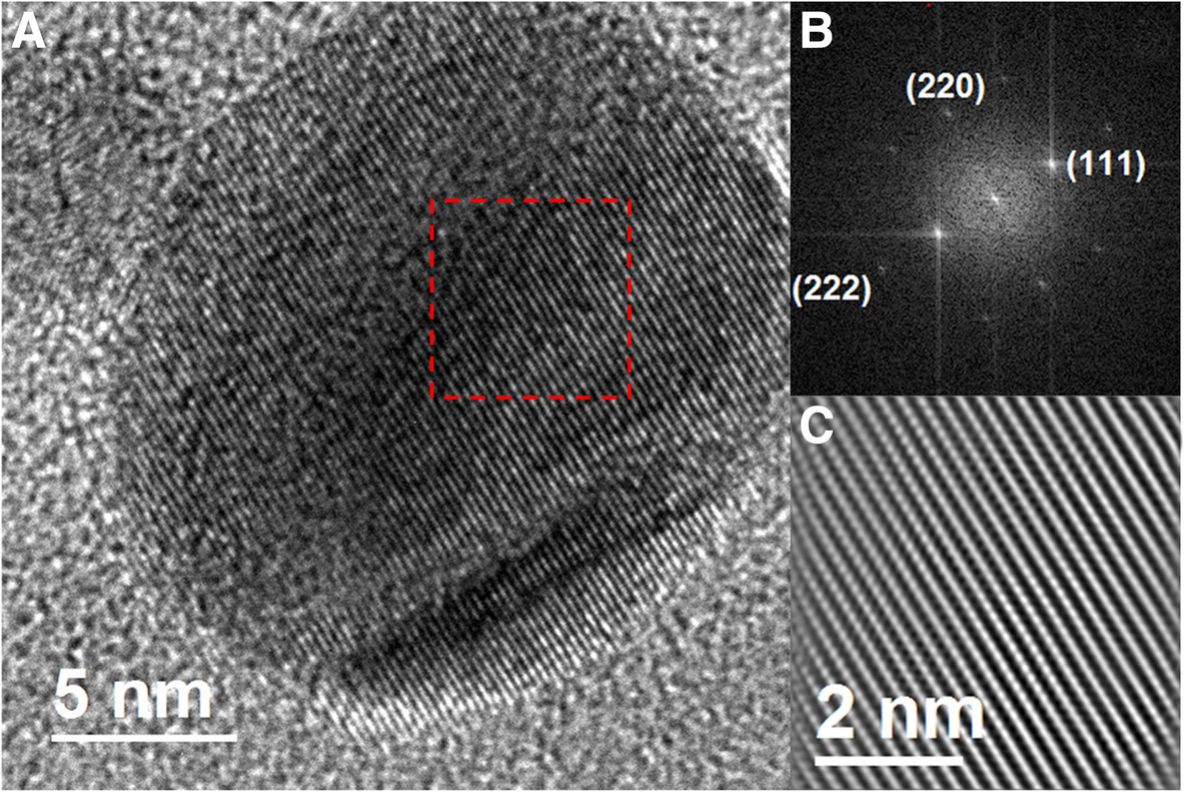
**HR-TEM images of a representative nanoparticl, with fcc structure.** HR-TEM image of a silver nanoparticle, the crystal planes correspond to a fcc structure **(A)** with its corresponding FFT plot **(B)**. The other figure **(C)** is an integrated image from the FFT plot. The reaction time was 96 h.

**Figure 7:**
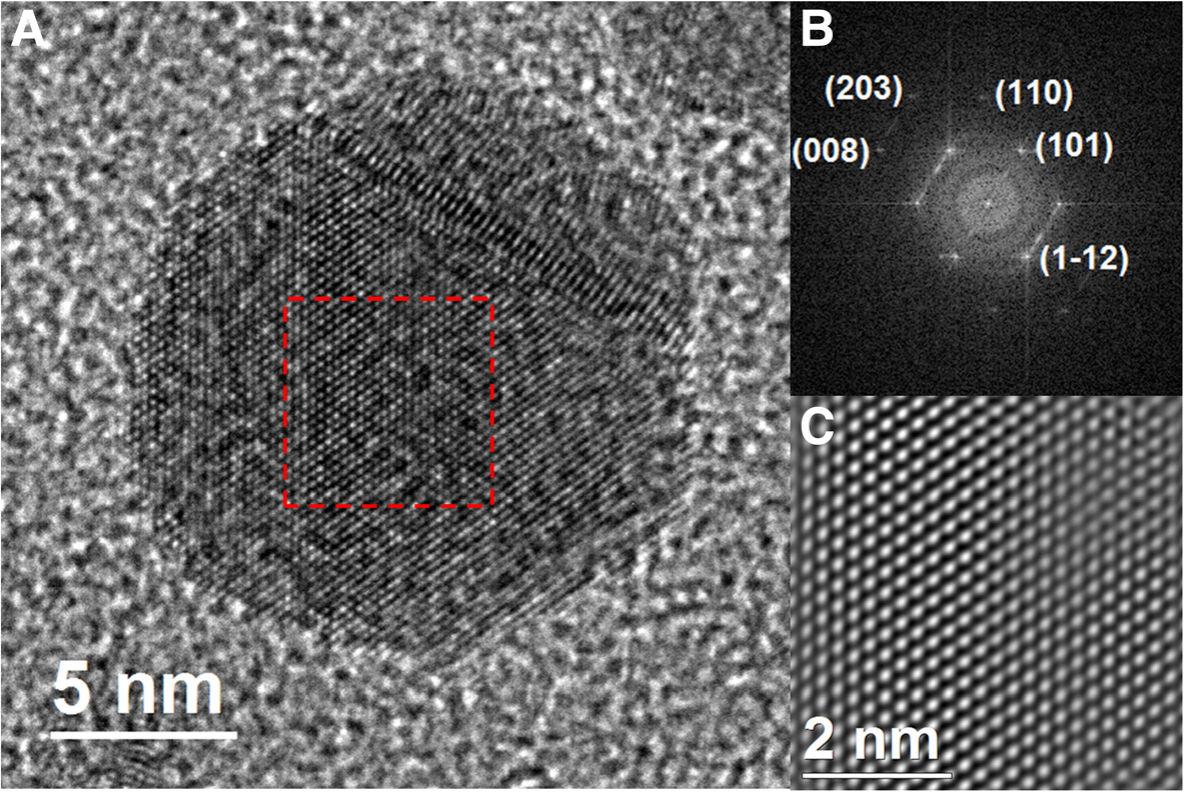
**HR-TEM images of a representative nanoparticle, with hexagonal (4H) structure.** HR-TEM image of a silver nanoparticle, the crystal planes correspond to a hexagonal (4H) structure **(A)** with its corresponding FFT plot **(B)**. The other figure **(C)** is an integrated image from the FFT plot. The reaction time was 96 h.

**Figure 8:**
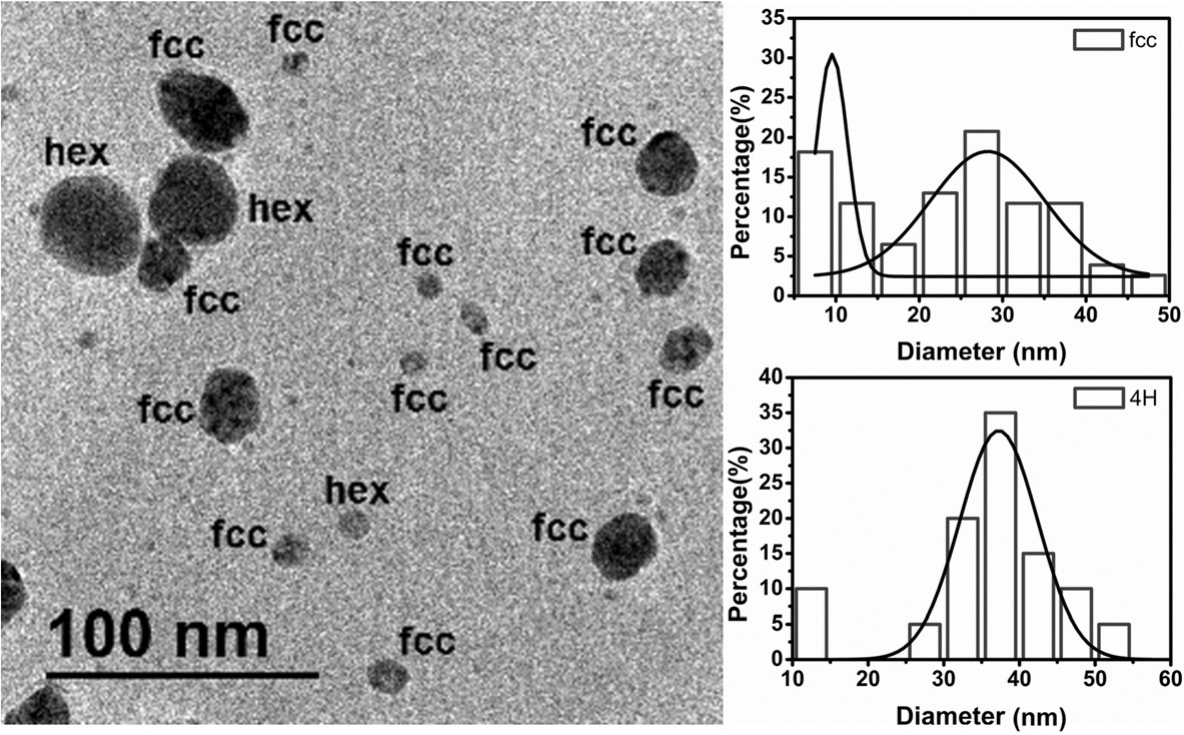
**TEM micrograph displaying both fcc and 4H nanoparticles.** The population histogram for each crystal structure is also displayed. The statistical analysis has been performed with 100 nanoparticles. The reaction time was 30 days.

The crystallographic analysis confirms that the nanoparticles are indeed silver crystals. For instance, in Figures 6 and 7, we show HR-TEM images of two representative nanoparticles, with the corresponding FFT plot. Very interestingly, these results show that the nanoparticle population has a combination of two kinds of crystal symmetries: face centered cubic (fcc) and hexagonal (4H). The prevalence rates of these geometries are 79% (fcc) and 21% (4H). We have computed the interplanar distances from the micrographs and the FFT plots. In the case of the fcc nanoparticles, the interplanar distances are *d*_1_ = 2.316 Å, *d*_2_ = 1.517 Å, and *d*_3_ = 1.159 Å. They are, respectively, associated with the planes (111), (220), and (222) corresponding to the fcc structure of a silver crystal. On the other hand, the interplanar distances for the 4H structure are *d*_1_ = 2.405 Å, *d*_2_ = 2.275 Å, *d*_3_ = 1.407 Å, *d*_4_ = 1.249 Å, and *d*_5_ = 1.149 Å, corresponding to the planes (101), (1-12), (110), (008), and (203) of a hexagonal 4H structure [61]. We have characterized the nanoparticle population for both the fcc and 4H structures, analyzing 100 particles. The results are shown in Figure 8. We observe that the fcc nanoparticles display two size populations: one with a small average diameter (around 10 nm) and a second one with a larger diameter (around 28 nm). On the other hand, the hexagonal nanoparticles have only one size population and larger diameters (around 38 nm). Note that the results shown in Figure 8 correspond to samples where the reaction time is of 30 days.

The observed features in the TEM, UV-Vis,, and visual observation experiments can be summarized and understood as follows. The polyphenols contained in the *R. hymenosepalus* extracts act effectively as reducing agents for the Ag^+^ ions due to their antioxidant activity. The reduction reaction promotes the nucleation and growth of nearly spherical Ag nanoparticles. As expected, the kinetics of nanoparticle formation, as well as the resulting nanoparticle populations, depends on the AgNO_3_ concentration. Higher silver nitrate concentrations yield more nanoparticles for reacting times of 24 h, because more material is available for the nanoparticle growth. However, when the reaction time is 96 h, two populations of nanoparticles are present. In this case, most of the silver atoms are accommodated in large nanoparticles.

## Conclusions

We have prepared silver nanoparticles using extracts of *R. hymenosepalus*, a plant abundantly found in North Mexico and in the south of the USA, as reducing agent. The results are very promising since the extract promotes the formation of nanoparticles at room temperature with a fast kinetics and with no harmful chemicals. Our method is easy to perform in a single step. NMR and UV-Vis spectroscopy experiments show that *R. hymenosepalus* is a plant rich in polyphenols, such as catechines and stilbenes, molecules that have antioxidant activity and are also found in plants like green tea and grapes. The same molecular mechanisms responsible of the antioxidant activity allow the use of these molecules as reducing agents and stabilizing effects for silver nanoparticles. The silver nanoparticles synthesized by this method are strong candidates for its use in biological systems. The diameter of the silver nanoparticles is in the range of 2 to 40 nm, as shown by TEM experiments. Interestingly, the silver nanoparticle population is composed of a mixture of face-centered cubic and hexagonal structures. The presence of the hexagonal crystal atypical structure 4H for silver nanoparticles was obtained by this method, opening a new route to study catalytical activity, antimicrobial properties, and the optical response of this nanomaterial.

## Competing interests

The authors declare that they have no competing interests.

## Authors' contributions

ERL, RIP, and REN carried out the experiments. ERL, RIP, REN, JT, RHU, and AM analyzed the data. CIP conducted the plate count experiments. ERL, RIP, JT, and AM developed the conceptual framework, and AM supervised the whole work. ERL, RIP, and AM drafted the paper. All authors read and approved the final manuscript.

## Supplementary Material

Additional file 1 **Dried roots of *Rumex hymenosepalus *(Figure S1). **^1^H NMR spectra of Rh in DMSO-d6 referenced to TMS **(Figure S2)**. Section of the 1H NMR spectra of the Rh extract **(Figure S3)**. Following section of the 1H NMR spectra of the Rh extract **(Figure S4)**. ^1^H NMR chemical shifts for the Rh extract (first column) as compared to those reported in the literature **(Table S1)**. Molecular structure of the catechin compounds found in the Rh extract **(Figure S5)**. Molecular structure of stilbene glycoside found in the Rh extract **(Figure S6)**. Composition of samples without Rh extract **(Table S2)**. UV-Vis spectrum of solutions without Rh extract **(Figure S7)**. Sterility test of **(A)** aerobic mesophilic bacteria and **(B)** mold and yeast **(Figure S8)**. EDS spectra for a silver nanoparticle **(Figure S9)**. Chemical analysis of the EDS results for a silver nanoparticle **(Table S3)**. Click here for file
