# Supplementary material for: Synthesis of silver nanoparticles using reducing agents obtained from natural sources (Rumex hymenosepalus extracts)
Source: Nanoscale Res Lett. 2013 Jul 10;8(1):318. doi: 10.1186/1556-276X-8-318 (PMC3716778; doi:10.1186/1556-276X-8-318)
Supplement: Additional file 1 — Dried roots of Rumex hymenosepalus (Figure S1). 1H NMR spectra of Rh in DMSO-d6 referenced to TMS (Figure S2). Section of the 1H NMR spectra of the Rh extract (Figure S3). Following section of the 1H NMR spectra of the Rh extract (Figure S4). 1H NMR chemical shifts for the Rh extract (first column) as compared to those reported in the literature (Table S1). Molecular structure of the catechin compounds found in the Rh extract (Figure S5). Molecular structure of stilbene glycoside found in the Rh extract (Figure S6). Composition of samples without Rh extract (Table S2). UV-Vis spectrum of solutions without Rh extract (Figure S7). Sterility test of (A) aerobic mesophilic bacteria and (B) mold and yeast (Figure S8). EDS spectra for a silver nanoparticle (Figure S9). Chemical analysis of the EDS results for a silver nanoparticle (Table S3). [file 1556-276X-8-318-S1.pdf]

Additional File 1

Synthesis of Silver Nanoparticles using reducing agents from Natural Sources (Rumex hymenosepalus extracts)

By Ericka Rodríguez-León et al

In this section we present more detail on some experimental results discussed in the main manuscript.

I. Picture of dried roots of Rumex hymenosepalus

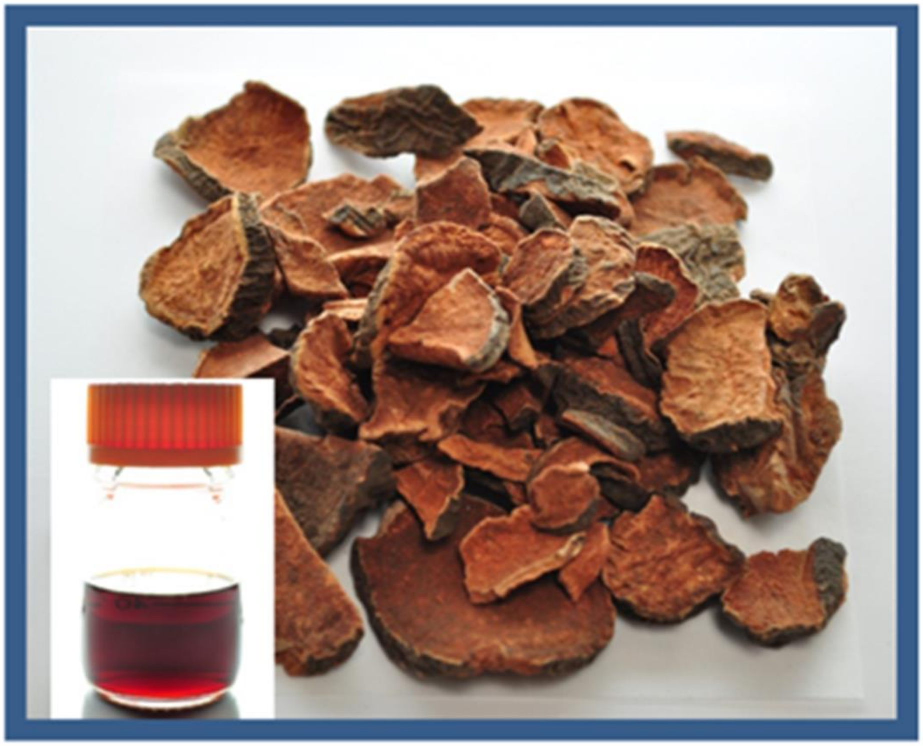

Figure S1.- Dried Roots of Rumex hymenosepalus. On inset extract solution of Rh after filtration.

In particular, we display some ^{1}H NMR spectra and the EDS results, as well as a table with the NMR chemical shifts and the chemical structures of the found polyphenol molecules. In the following, Rh stands for *Rumex hymenosepalus*.

II. ^{1}H NMR results

Figures 2, 3 and 4 display the ^{1}H NMR spectrum of the Rh extract. Figure S2 is the full spectrum while figures S3 and S4 are sections of it. The observed chemical shifts correspond to polyphenol molecules, as shown in Table S1.

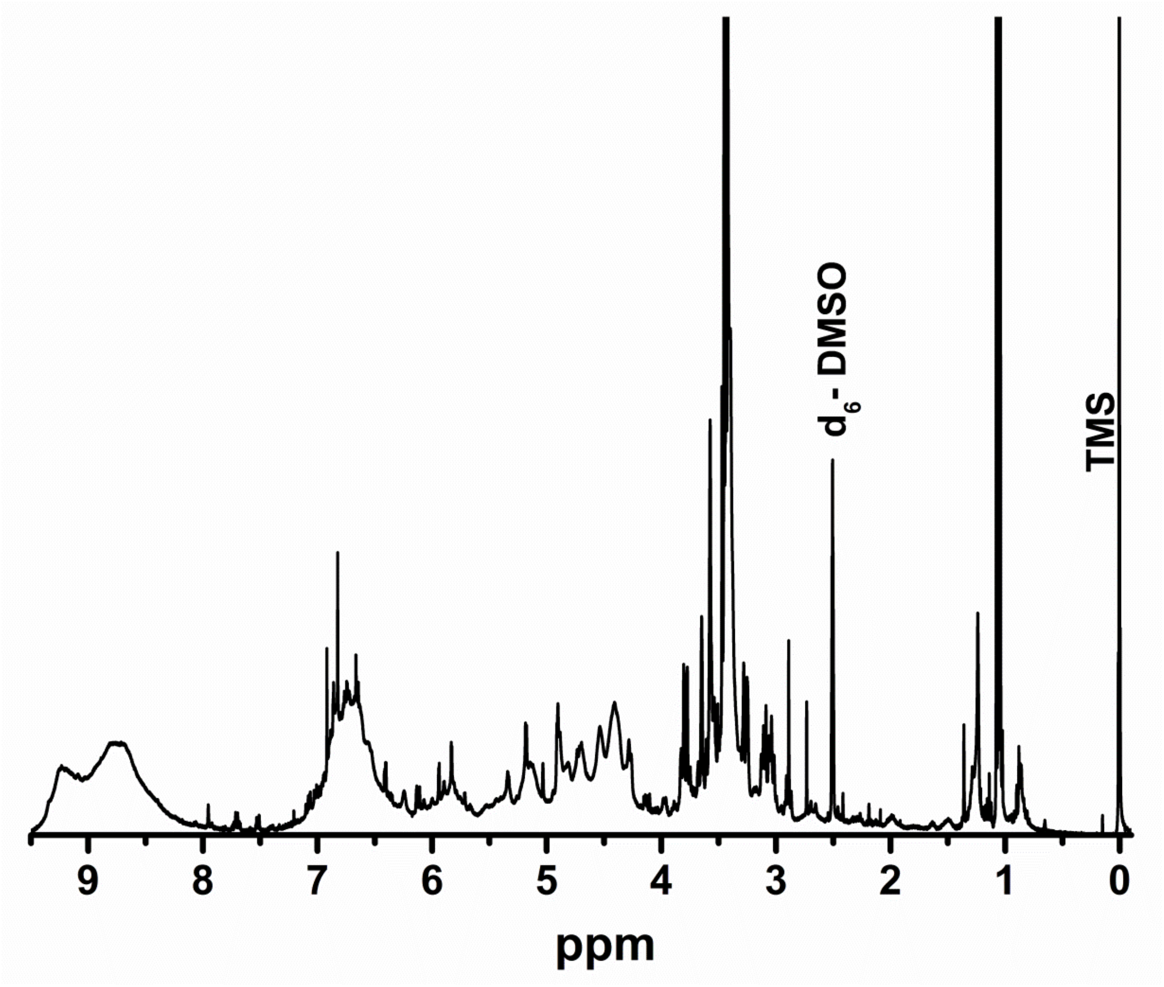

Figure S2.- ^{1}H NMR spectra of Rh in DMSO-d_6 referenced to TMS.

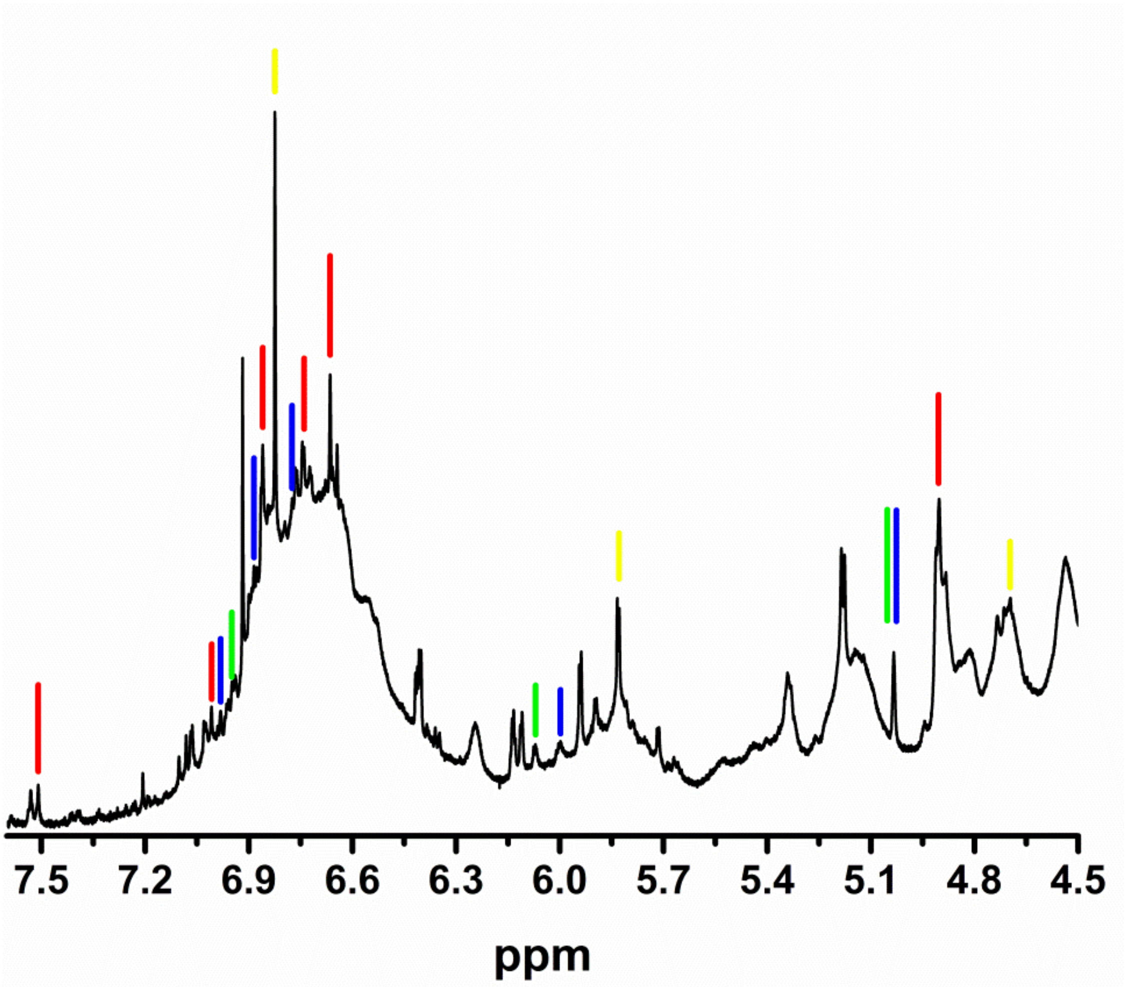

Figure S3.- Section of the ^{1}H NMR spectra of the Rh extract. The colored lines in the chemical shifts correspond to polyphenol molecules: stilbene glycoside (red), epicatechin (yellow), epicatechin gallate (blue) and epigallocatechin gallate (green).

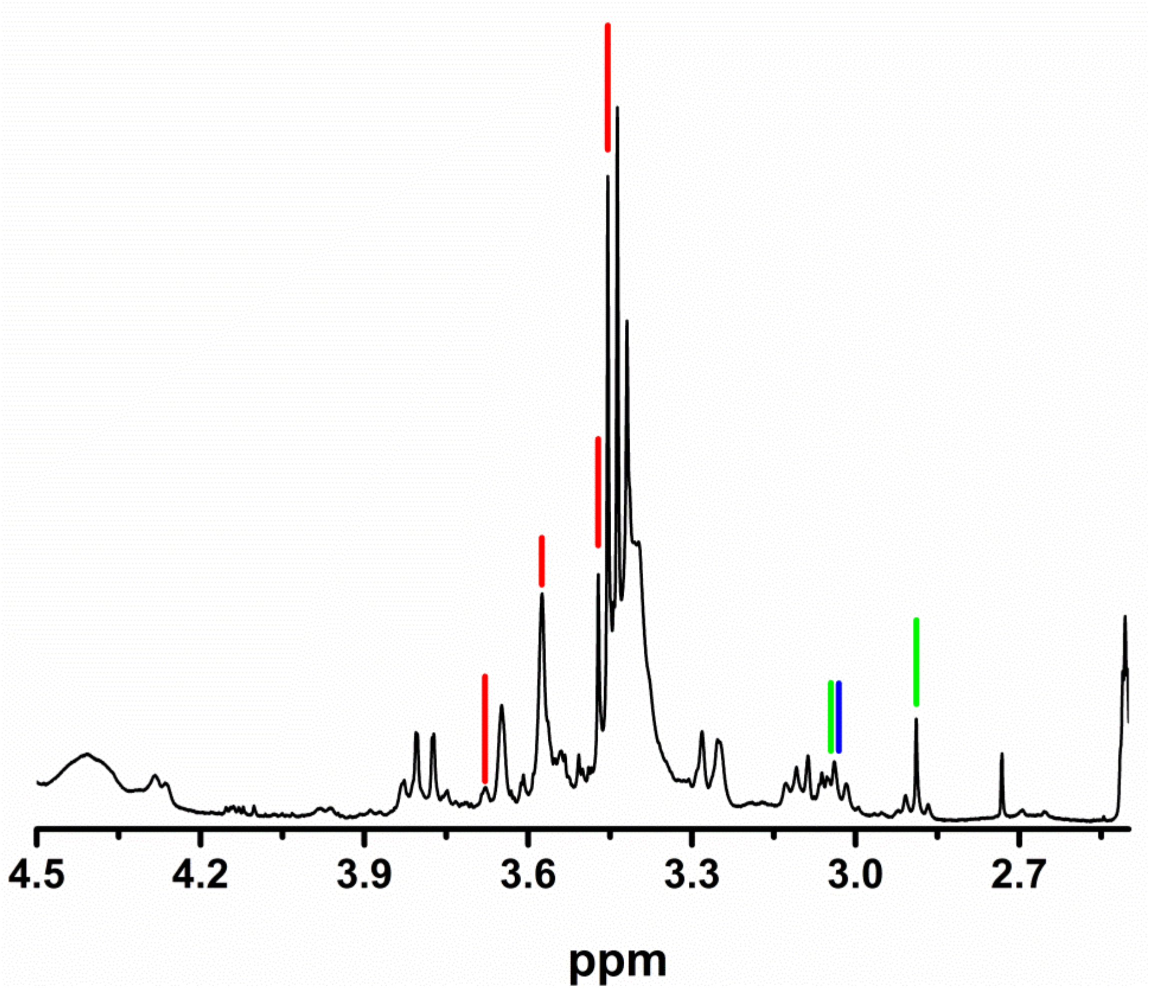

Figure S4.- Following section of the ^{1}H NMR spectra of the Rh extract. The colored lines in the chemical shifts correspond to polyphenol molecules: stilbene glycoside (red), epicatechin gallate (blue) and epigallocatechin gallate (green).

In Table S1 we display the observed chemical shifts in the Rh extract, and compare them to those of different polyphenol molecules reported in the literature: stilbene glycoside [1], epicatechin (EC) [3], epicatechin gallate (ECG) [2] and epigallocatechin gallate (EGCG) [2]. Reference [3] is the web page of Spectral Database for Organic Compounds, SDBS. Note that the agreement is remarkably good.

Chemical Shift extract	Stilbene Glycoside [1]	EGCG [2]	ECG [2]	EC [3]
7.50	7.49			
7.00	6.99			
6.98			6.99	
6.94		6.95		
6.88			6.88	
6.86	6.85			
6.82				6.821
6.77			6.78	
6.74	6.74			
6.66	6.66			
6.07		6.03	6.03	
5.82				5.821
5.03		5.03	5.09	
4.90	4.90			
4.73	4.75			
4.69				4.66
3.92				3.928
3.90	3.90			
3.71	3.71			
3.68	3.68			
3.57	3.58			
3.56	3.56			
3.47	3.46			
3.43	3.44			

3.03		3.03	3.03	
2.88		2.89		
2.41				2.4
1.21	1.22			

Table S1.- ^{1}H NMR chemical shifts for the Rh extract (first column) as compared to those reported in the literature for stilbene glycoside, epigallocatechin gallate (EGCG), epicatechin gallate (ECG) and epicatechin (EC).

III. Polyphenol molecular structures

In figures S5 and S6 we present the chemical structures of some of the molecules present in the Rh extract.

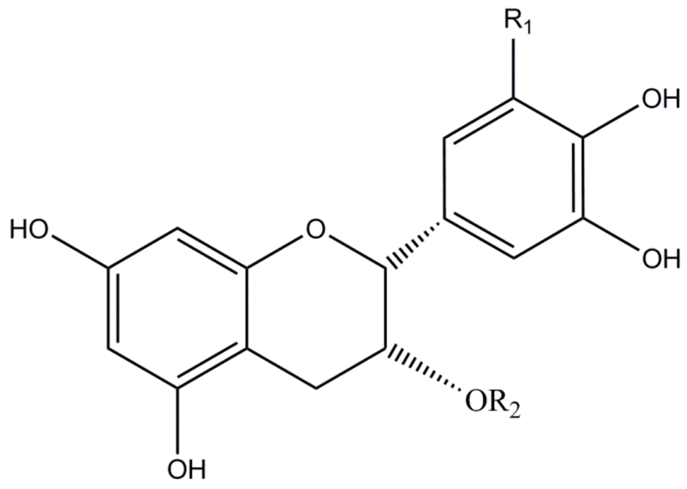

ec, R_1=H, R_2=H ecg, R_1=H, R_2=gallate egcg, R_1=OH, R_2=gallate

gallate=

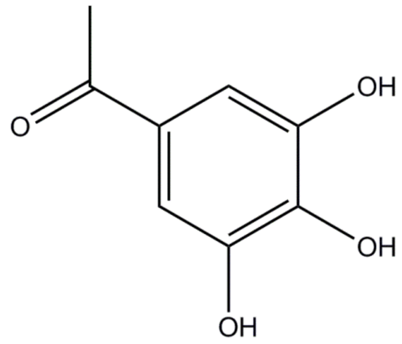

Figure S5.- Molecular structure of the catechin compounds found in the Rh extract.

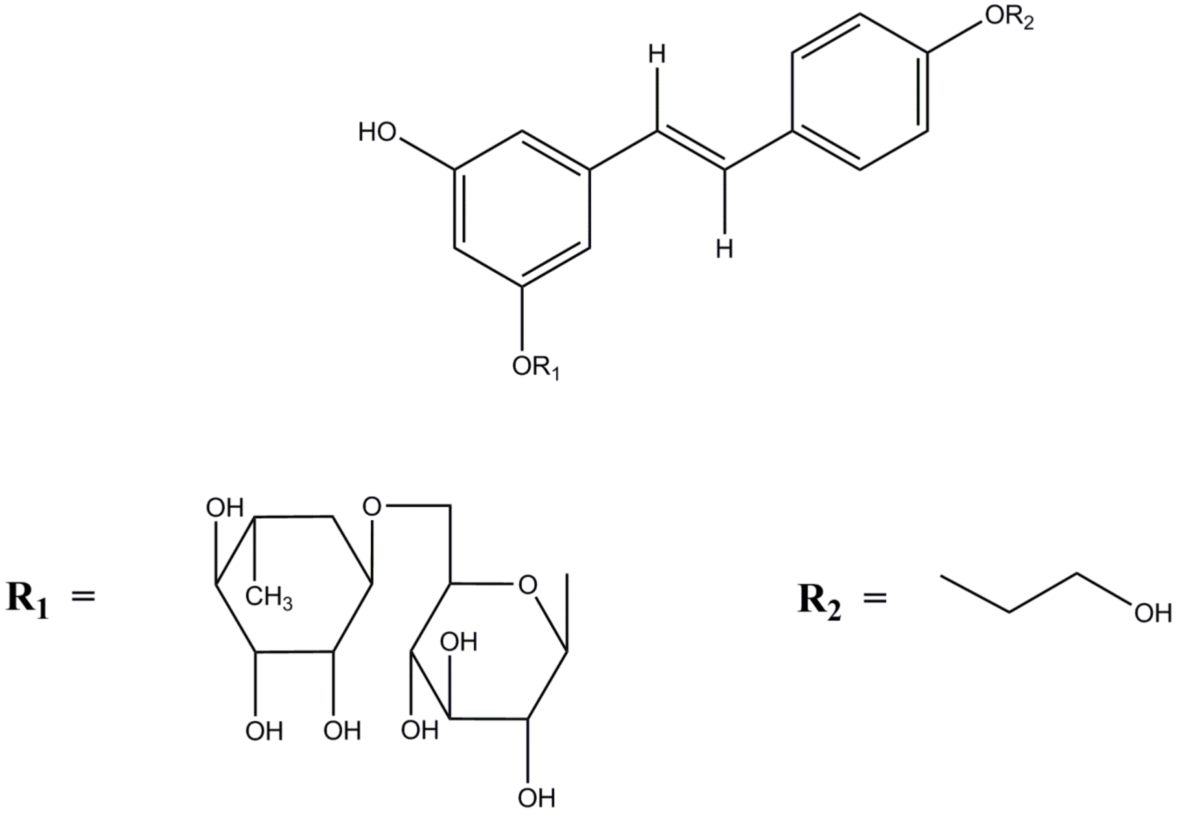

Figure S6.- Molecular structure of stilbene glycoside found in the Rh extract. This molecule has also been found in the *Terminalia sericeae* tree.

IV. Negative control using solutions of $AgNO_3$ and ethanol without Rh extract.

Sample	***Vol. $AgNO_3$ 0.1M***	***Vol. Sol. 70:30 v/v EtOH/Water***	***Vol. EtOH***
**SA**	100µL	200 µL	3700 µL
**SB**	600 µL	200 µL	3200 µL

Table S2.- Composition of samples without Rh extract.

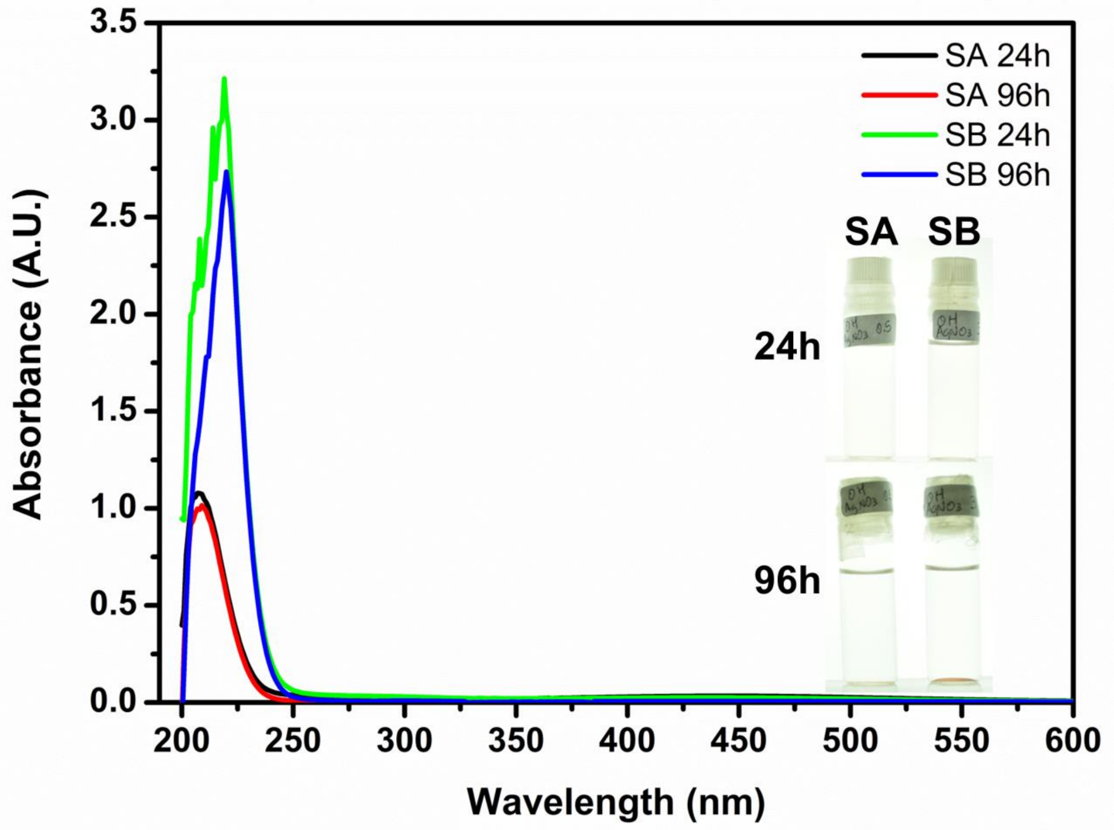

Figure S7.- UV-Vis spectrum of solutions without Rh extract. This is the negative control for the synthesis of silver nanoparticles.

V. Negative control for abiotic synthesis of silver nanoparticles

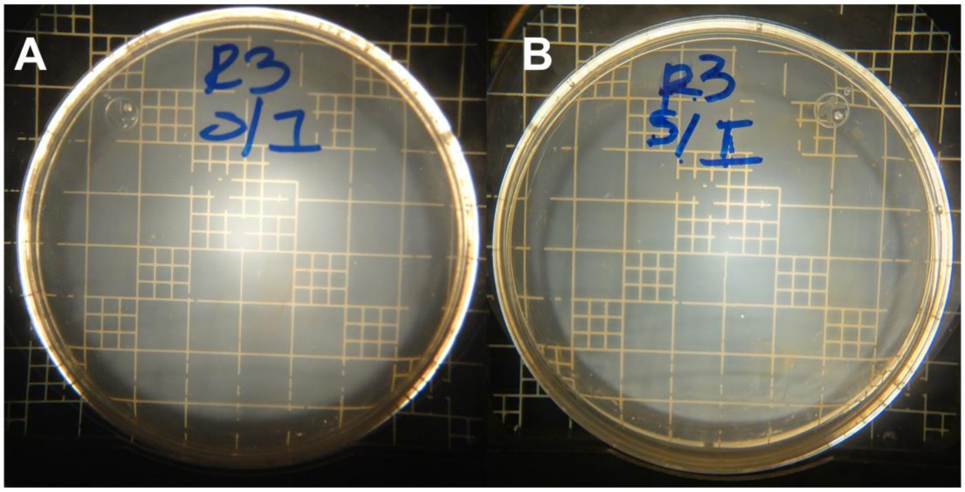

Figure S8.- Sterility test of A)aerobic mesophilic bacteria and B) mold and yeast. Both samples are free of microorganisms.

VI. Energy Dispersive X-Ray spectroscopy (EDS) results

Figure S9 and Table S3 show the EDS results.

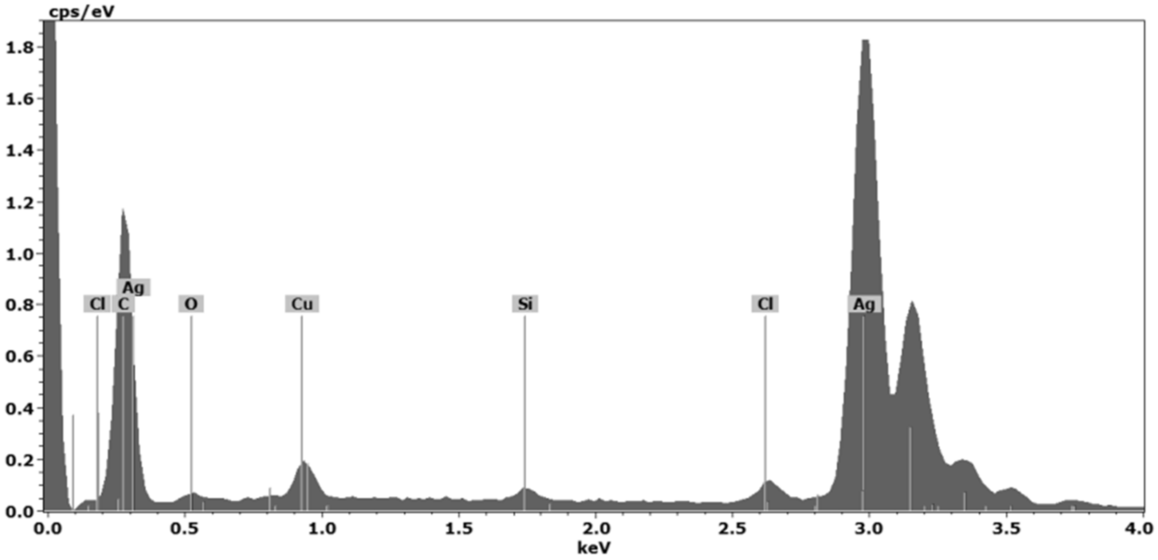

Figure S9.- EDS spectra for a silver nanoparticle. Note the peaks corresponding to silver.

E1	AN	Series	unn. C [wt. %]	Norm. C [wt. %]	Atom C [at. %]	Error (1 Sigma) [wt. %]
Ag	47	K-series	99.05	99.05	96.88	3.30
K	19	K-series	0.66	0.66	1.78	0.06
O	8	K-series	0.11	0.11	0.73	0.04
S	16	K-series	0.17	0.17	0.56	0.04
Mg	12	K-series	0.01	0.01	0.04	0.03
C	6	K-series	0.00	0.00	0.00	0.00
Si	14	K-series	0.00	0.00	0.00	0.00
Cl	17	K-series	0.00	0.00	0.00	0.00
Cu	29	K-series	0.00	0.00	0.00	0.00
N	7	K-series	0.00	0.00	0.00	0.00
		Total:	100.00	100.00	100.00	

Table S3.- Chemical analysis of the EDS results for a silver nanoparticle. The particle is mainly composed of silver (96.88%) but trace amounts of other elements are also present. These elements are often found in plant extracts.

References

1. Joseph CC, Moshi MJ, Innocent E, and Nkunya MHH: **Isolation of a Stilbene Glycoside and other Constituents of Terminalia sericeae.** Afr J Tradit Complementary and Alternative Medicine 2007, 4: 383–386.
2. Sivaraman SK, Elango I, Kumar S and Santhanam V: **A green protocol for room temperature synthesis of silver nanoparticles in seconds.** Current Science 2009, 97:1055-1059.
3. http://sdbs.riodb.aist.go.jp/sdbs/cgi-bin/cre_index.cgi
